# Interactions between halotolerant nitrogen-fixing bacteria and arbuscular mycorrhizal fungi under saline stress

**DOI:** 10.3389/fmicb.2024.1288865

**Published:** 2024-03-13

**Authors:** Chao Ji, Yuhan Ge, Hua Zhang, Yingxiang Zhang, Zhiwen Xin, Jian Li, Jinghe Zheng, Zengwen Liang, Hui Cao, Kun Li

**Affiliations:** ^1^College of Seed and Facility Agricultural Engineering, Weifang University, Weifang, China; ^2^Key Laboratory of Biochemistry and Molecular Biology in University of Shandong Province, Weifang University, Weifang, China; ^3^Mountain Tai Forest Ecosystem Research Station of State Forestry and Grassland Administration, Shandong Agricultural University, Tai’an, China; ^4^State Forestry and Grassland Administration Key Laboratory of Silviculture in Downstream Areas of the Yellow River, Shandong Agricultural University, Tai’an, China; ^5^College of Biology and Oceanography, Weifang University, Weifang, China; ^6^Shandong Institute of Pomology, Tai’an, China; ^7^Research Center for Forest Carbon Neutrality Engineering of Shandong Higher Education Institutions, Tai’an, Shandong, China; ^8^Key Laboratory of Ecological Protection and Security Control of the Lower Yellow River of Shandong Higher Education Institutions, Tai’an, Shandong, China

**Keywords:** salt stress, nitrogen fertilization, halotolerant nitrogen-fixing bacteria, arbuscular mycorrhizal fungi, plant growth-promoting rhizobacteria

## Abstract

**Background and aims:**

Soil salinity negatively affects crop development. Halotolerant nitrogen-fixing bacteria (HNFB) and arbuscular mycorrhizal fungi (AMF) are essential microorganisms that enhance crop nutrient availability and salt tolerance in saline soils. Studying the impact of HNFB on AMF communities and using HNFB in biofertilizers can help in selecting the optimal HNFB-AMF combinations to improve crop productivity in saline soils.

**Methods:**

We established three experimental groups comprising apple plants treated with low-nitrogen (0 mg N/kg, N0), normal-nitrogen (200 mg N/kg, N1), and high-nitrogen (300 mg N/kg, N2) fertilizer under salt stress without bacteria (CK, with the addition of 1,500 mL sterile water +2 g sterile diatomite), or with bacteria [BIO, with the addition of 1,500 mL sterile water +2 g mixed bacterial preparation (including *Bacillus subtilis* HG-15 and *Bacillus velezensis* JC-K3)].

**Results:**

HNFB inoculation significantly increased microbial biomass and the relative abundance of beta-glucosidase-related genes in the rhizosphere soil under identical nitrogen application levels (*p* < 0.05). High-nitrogen treatment significantly reduced AMF diversity and the relative abundance of beta-glucosidase, acid phosphatase, and urea-related genes. A two-way analysis of variance showed that combined nitrogen application and HNFB treatment could significantly affect soil physicochemical properties and rhizosphere AMF abundance (*p* < 0.05). Specifically, HNFB application resulted in a significantly higher relative abundance of *Glomus-MO-G17-VTX00114* compared to that in the CK group at equal nitrogen levels.

**Conclusion:**

The impact of HNFB on the AMF community in apple rhizospheres is influenced by soil nitrogen levels. The study reveals how varying nitrogen levels mediate the relationship between exogenous HNFB, soil properties, and rhizosphere microbes.

## 1 Introduction

Approximately 20% of the world’s arable land is currently at risk of salinity, and this percentage is steadily increasing by 10% each year, posing a considerable challenge to agricultural production and contributing to land degradation (Farooq et al., 2021). Apple trees are highly resistant to salinity and alkali stress, making them the preferred fruit-tree species for the efficient development and utilization of saline-alkali land in the Yellow River Basin. However, nitrogen deficiency is a major cause of restricted apple growth in saline soils. Typically, crop yield is improved with the application of nitrogen. However, excessive nitrogen application does not always result in a continuous yield increase; it reduces nitrogen-use efficiency and leads to environmental problems (Grassini et al., 2013; Faostat, 2016). Thus, it is important to consider that using inorganic nitrogen fertilizers can increase nutrient amounts and soil salinity, which may damage plants instead of promoting growth (Jha et al., 2012). In apple cultivation on saline-alkali lands, one potential solution to address this issue is to develop efficient, sustainable, and environmentally friendly biological nitrogen fertilizers that can partially replace chemical nitrogen fertilizers (Chen et al., 2023a).

Many studies have confirmed the importance of microbial inoculants in achieving higher crop yields, improving crop quality and soil fertility, and deepening our understanding of the mechanisms of interactions between certain bacterial and plant strains in specific ecosystems (Primieri et al., 2021; Liu et al., 2022). However, there are still some problems with the application of nitrogen-fixing bacteria under natural conditions: (1) several nitrogen-fixing bacterial strains struggle to colonize saline soils for extended periods, resulting in unstable nitrogen fixation effects. Additionally, there has been limited research on the screening methods, application, and mechanism of action of salt-tolerant nitrogen-fixing bacteria, also known as halotolerant nitrogen-fixing bacteria (HNFB). (2) While several studies have focused on the growth-promoting effects of exogenously inoculated plant growth-promoting rhizobacteria (PGPR) on host plants (Thirkell et al., 2020), only a few have examined the synergistic effects of PGPR with other rhizosphere microorganisms on saline soils and crops, especially beneficial flora with unique abilities. In recent years, there has been a trend toward developing compound bacterial fertilizers comprising two or more bacterial strains instead of single-strain fertilizers, and a shift from fuzzy decision support systems to clear decision support systems. For example, in maize stems, xylem selectively recruited conserved microorganisms dominated by γ-proteobacteria, and the combination of *Klebsiella* var*iicola* MNAZ1050 and *Citrobacter* sp. MNAZ1397 increased nitrogen accumulation in maize by 11.8% (Zhang et al., 2022).

Halotolerant PGPRs with 1-aminocyclopropane-1-carboxylic acid (ACC) deaminase activity can reduce plant ethylene accumulation, prevent oxidative stress, promote plant growth, and regulate the rhizosphere microbial community structure. Consequently, halotolerant PGPRs have become a valuable microbial resource for promoting crop growth in saline-alkali soils (Ji et al., 2022a; Li et al., 2023). Among the beneficial microorganisms in saline soils, ascomycetous fungi (AMF) can form a symbiotic relationship with most crops, improving soil nutrient acquisition, promoting plant growth and water absorption, and initiating defense responses in host plants (Bennett and Groten, 2022). Although AMF may not prove to be a “sustainable savior” in agroecosystems (Thirkell et al., 2017), they have the potential to help crops assimilate nutrients (Thirkell et al., 2020). Currently, there is a lack of research on HNFB-mediated AMF community responses.

Changes in the core strains in the rhizosphere of crops can alter the types and quantities of metabolites and affect the interaction network of the entire community (Coyte et al., 2015). For example, Niu et al. (2017) constructed a synthetic community composed of seven bacteria and found that in the absence of *Enterobacter cloacae*, the abundance of *Brevibacterium parvum* increased, while other species disappeared from the community, demonstrating the importance of key species in the microbiome. Therefore, analyzing the composition of core microorganisms is necessary for accurately regulating the rhizosphere microbial community, improving microbial community function, and elucidating the mechanisms of microbial community-plant interactions (Hogle et al., 2023). However, because chemical nitrogen fertilizers and nitrogen-fixing bacteria coexist in the agricultural sector, they may interact with each other, with uncertain consequences for the soil–microbe system (Tian et al., 2017); whether the coexistence of chemical nitrogen fertilizers and nitrogen-fixing bacteria affects the core strains remains unknown.

In this study, we inoculated the rhizosphere of apple trees in saline land with a compound flora composed of two salt-tolerant strains that can stably colonize saline land, exhibit ACC deaminase activity, and demonstrate high efficiency in synergistic nitrogen fixation. Additionally, we examined the effects of compound inoculation on the apple plants and the rhizosphere AMF communities under three nitrogen application levels. We proposed and tested the following two hypotheses: (1) the AMF community in the rhizosphere of apple trees in saline-alkali soil is unique, and excessive nitrogen application has a negative effect on the structure and function of the AMF community; (2) exogenous HNFB positively affects the structure and function of the AMF community by influencing one or more core rhizosphere AMF species.

## 2 Materials and methods

### 2.1 HNFB strains and culture media

The microbial inoculant consisted of a mixture of *Bacillus subtilis* HG-15 and *Bacillus velezensis* JC-K3 strains. The 16S rDNA sequences of these two strains were deposited in the NCBI database under accession number MN689681 and MT605169. Both strains have been shown to possess efficient nitrogen fixation ability, antagonistic activity, and other growth-promoting characteristics in our previous studies (Ji et al., 2021, 2022b). The nitrogen fixation activity of the HG-15 strain is 24.30 ± 0.75 mg N/g glucose, while that of the JC-K3 strain is 30.25 ± 0.42 mg N/g glucose. The ACC deaminase activity of the HG-15 strain is 14.816 ± 0.965 μmol/(mg h), while that of the JC-K3 strain is 18.10 ± 0.97 μmol/(mg h). Luria–Bertani liquid medium was used as the seed and fermentation medium. When the spore formation rate in the fermentation liquid exceeded 95%, diatomite sterilized at 121°C for 20 min was added at a concentration of 10% to the fermented liquid. The bacteria were allowed to adsorb onto the diatomite. The suspension was then centrifuged at 3,100 × *g* for 20 min. The supernatant was discarded, and the sediment was stored at −40°C for 48 h before being placed in a lyophilizer (Labconco FreeZone® Plus 4.5 L; Kansas City, MO, USA) and treated at −48°C and 9 Pa for 48 h (Ji et al., 2020). The densities of the HG-15 and JC-K3 strains in the resulting solid microbial agents were 451 × 10^8^ CFU/g and 498 × 10^8^ CFU/g, respectively. The two bacterial preparations were diluted to a concentration of 20 × 10^8^ CFU/g with sterile diatomite, and then mixed in a 1:1 ratio for later use.

### 2.2 Experimental design

The experiment was conducted in the Weifang Economic Development Zone (119°3′30′′E, 36°48′14′′N), Shandong Province, China, in 2022. A four-year-old dwarf rootstock M9T337 grafted Fuji apple (Red Fuji) plant was selected as the test material [electrical conductivity (EC) = 671 s/cm, pH = 7.6, 14.51 g/kg soil organic matter, 59.25 mg/kg soil available nitrogen, 21.46 mg/kg soil available phosphorus (by Olsen P test), and 122.17 mg/kg soil exchangeable potassium]. Before planting, all seedlings were fertilized with urea (46% N) at three different concentrations: 0 mg N/kg (N0, low nitrogen level), 200 mg N/kg (N1, normal nitrogen level), and 300 mg N/kg (N2, high nitrogen level), which corresponded to 0, 240, and 360 kg N/ha of field fertilizer (urea), respectively. Simultaneously, 10 g of potassium sulfate (containing 50% K_2_O) and 17 g of calcium superphosphate (containing 14% P_2_O_5_) were applied per plant. Apple plants with strong and uniform growth were selected and planted in pots in mid-March, with one plant per pot. The pots and soil used in this experiment were not sterilized. After 14 days of plant colonization, the three nitrogen application levels were established: without bacteria (CK, with the addition of 1,500 mL sterile water +2 g sterile diatomite) or with bacteria (BIO, with the addition of 1,500 mL sterile water +2 g mixed bacterial preparation). This resulted in a total of six groups, each containing 12 pots. The experimental plants were arranged randomly, while the other experiments were conducted according to standard field procedures. Samples were taken at the flower bud morphological differentiation stage in July 2022. This experiment was repeated thrice.

### 2.3 Rhizosphere soil sampling and analysis

Soil pH and EC values were analyzed using digital pH (FE20) and EC (FE930) meters (Mettler Toledo, Switzerland), respectively. The soil-water ratios used for analysis were 1:2.5 and 1:5. The organic matter content of the soil was determined using the method described by Aj and Black (1934). The total nitrogen content in the soil (soil N) was determined using the Bremner (2009) method.

Microbial biomass carbon (MBC) and microbial biomass nitrogen (MBN) contents were determined using the chloroform fumigation-K_2_SO_4_ extraction method (Brookes et al., 1985; Vance et al., 1987), while microbial biomass phosphorus (MBP) content was determined using the chloroform fumigation-NaHCO_3_ extraction method (Brookes et al., 1982).

### 2.4 DNA extraction and polymerase chain reaction (PCR)-based amplification

Soil genomic DNA was extracted from 0.5 g of soil using a FastDNA SPIN Kit for Soil (MP Biomedicals, Irvine, CA, USA) according to the manufacturer’s instructions. The DNA quality was examined using 1.0% agarose gel electrophoresis, and the DNA concentration was quantified using a NanoDrop 2000 UV–Vis spectrophotometer (Wilmington, USA) (Zeng et al., 2021). Nested PCR was conducted to amplify specific fragments of the AMF 18S rRNA gene. The first PCR reaction consisted of a 20-μL mixture containing 1 μL of genomic DNA (approximately 10 ng), 2 μL of 2.5 mM dNTPs, 0.4 μL of FastPfu DNA Polymerase (5 U/μL), 0.4 μL of each primer [10 μM; AML1 (5′-ATCAACTTTCGATGGTAGGATAGA-3′)/AML2 (5′-GAACCCAAACACTTTGGTTTCC-3′) primer pair], 4 μL of 5-fold Fastpfu DNA Buffer (Takara, Dalian, China), and molecular-grade water. The products from the first PCR (approximately 10 ng used as the template) were then amplified in a second PCR reaction using the primers AMV4.5NF (5′-AAGCTCGTAGTTGAATTTCG-3′) and AMDGR (5′-CCCAACTATCCCTATTAATCAT-3′), following the same protocol as the first PCR step. The thermal cycling conditions for both PCR steps were as follows: initial denaturation at 95°C for 3 min, 27 cycles of denaturation at 95°C for 30 s, annealing at 55°C for 30 s, elongation at 72°C for 45 s, and final elongation at 72°C for 10 min. The PCR products were extracted using 2% agarose gels, purified with an AxyPrep DNA Gel Extraction Kit (Axygen, Union City, CA USA) according to the manufacturer’s protocol, and quantified with a QuantiFluor ST instrument (Promega, Madison, WI, USA).

### 2.5 Illumina MiSeq and bioinformatics analyses

Quantified and purified PCR products were sent to Majorbio BioPharm Technology Co. Ltd. (Shanghai, China) for sequencing using the Illumina MiSeq PE300 platform (San Diego, CA, USA). The raw sequences were deposited in the NCBI Sequence Read Archive (SRA) database (Accession ID PRJNA999929). The forward and reverse raw sequences were merged using FLASH (Mago and Salzberg, 2011) by overlapping paired-end reads with a required overlap length of >10 base pairs (bp) and quality controlled using Trimmomatic software (Bolger et al., 2014). Low-quality sequences (average quality score < 20) containing ambiguous bases, sequences without valid primer or barcode sequences, and sequences with a read length < 50 bp were excluded. The permitted maximum error ratio of the overlapping sequences was 0.2, which was used as the basis for screening overlapping sequences.

Non-repeating sequences were then extracted, and individual sequences that did not repeat were removed using Usearch 7.0 (Edgar, 2013). The sequences were subsequently clustered into operational taxonomic units (OTUs) with a 97% similarity cut-off using the QIIME software (Caporaso et al., 2010). After sequences were clustered, the taxonomy of each OTU was classified from the domain level to the OTU level using the RDP Classifier algorithm against the MaarjAM database (Maarjam 081) (ÖPik et al., 2010), with a default confidence threshold of 0.7.

### 2.6 Real-time quantitative reverse transcription PCR (qRT-PCR) analysis

The PCR products were purified and then ligated into a pMD18 vector using a pMD™ 18-T Vector Cloning Kit (TaKaRa Bio Inc.). Plasmid extraction and purification were performed using a MiniBEST Plasmid Purification Kit Ver. 4.0 (TaKaRa Bio Inc.). The concentration and purity of the plasmid were determined using a NanoDrop 2000 microspectrophotometer (Thermo Fisher Scientific, Waltham, MA, USA). After determining the plasmid copy number, the preparation was serially diluted to prepare 10^1^ to 10^8^ copies. A standard curve (*R*^2^ = 0.99), plotting the logarithm of the initial amount of template DNA as the abscissa and the Ct value of each diluted sample during the PCR reaction as the ordinate, was used to establish an amplification efficiency of 90–100%. The primers used for quantitative PCR (qPCR) amplification of the nitrogen-fixing bacteria were PolF (TGCGAYCCSAARGCBGACTC) and PolR (ATSGCCATCATYTCRCCGGA), while AM fungi were amplified using the AMV4.5NF and AMDGR primers. qPCR amplifications were performed using an ABI 7900 (USA) fluorescence quantitative PCR thermocycler using 15-μL reaction systems containing 7.5 μL × 2 SYBR Premix Ex Taq II, 0.3 μL × 50 ROX Reference Dye, 0.6 μL 10 μmol·L^−1^ pre-primer, 0.6 μL 10 μmol·L^−1^ post-primer, 2 μL DNA template, and 4.0 μL double-distilled water (ddH_2_O). The amplification program comprised an initial pre-denaturation at 95°C for 30 s, followed by 40 cycles of chain cleavage at 95°C for 5 s, annealing at 62°C for 30 s, extension at 72°C for 60 s, and signal acquisition at 83°C for 10 s. All samples were analyzed in triplicate.

### 2.7 Statistical analyses

Data analysis was performed using IBM SPSS 19.0 (IBM, Armonk, NY, USA). The plant and soil parameters followed a normal distribution, and Student’s *t*-test and one-way analysis of variance (ANOVA) were used to compare differences among plant parameters (*p* < 0.05). Interaction between nitrogen application and bacterial addition was detected using a two-way ANOVA. Redundancy analysis (RDA) was conducted to examine the relationships between the relative abundance of fungal and AMF taxa and the chemical properties of soil samples, using Canoco 4.5.1 (Microcomputer Power, Ithaca, NY, USA). The non-parametric factorial Kruskal–Wallis sum-rank test of the LEfSe tool was used to identify and detect the characteristics of groups exhibiting significant differences in abundance. LEfSe utilizes linear discriminant analysis (LDA) to estimate the effect of the abundance of each component (species) on the differences.

## 3 Results

### 3.1 Effects of nitrogen fertilizer and exogenous HNFB on soil chemical properties

Among the different nitrogen application treatments, the MBP and MBC were significantly higher in the BIO + N1 treatment group than in the BIO + N0 group (by 28.08 and 6.25%, respectively) and the BIO + N2 group (by 36.85 and 82.68%, respectively) (*p* < 0.05). The MBN content in the BIO + N1 treatment group was significantly higher than in the BIO + N2 treatment group (by 51.23%) (*p* < 0.05). The EC and pH values of the BIO + N0 and BIO + N2 treatment groups were significantly higher than those of the BIO + N1 group (*p* < 0.05). For the BIO treatment, as the concentration of applied nitrogen increased, MBN and MBP first increased and then decreased. Moreover, the MBC/MBN ratio decreased gradually, with values of 8.24, 7.27, and 6.02 for the three nitrogen application levels, respectively, (Supplementary Figure S1).

The MBC of the CK + N2 group was significantly lower than that of the CK + N0 group (by 39.07%) and the CK + N1 group (by 38.97%) (*p* < 0.05). The MBN of the CK + N1 group was significantly higher than that of the CK + N0 group (by 24.61%) and the CK + N2 group (by 112.68%) (*p* < 0.05). The EC of the CK + N2 group was significantly higher than that of the CK + N0 group (by 3.13%) and the CK + N1 group (by 9.65%) (*p* < 0.05). In the CK treatment groups, MBN first increased and then decreased with increasing nitrogen application levels. The MBC/MBN ratio exhibited a trend of initially decreasing and then increasing, with values of 8.46, 6.78, and 8.81 for the three nitrogen application levels, respectively. Nitrogen application did not result in significant differences in MBP or pH in the CK treatment groups (Supplementary Figures S1C,E). Under the same nitrogen level, the MBP, MBC, and MBN levels in the BIO group were significantly higher than those in the CK group (*p* < 0.05). Additionally, the EC level in the CK group was significantly higher than that in the BIO group (*p* < 0.05). There was no significant difference in the pH levels between the BIO and CK groups. The soil N content of the BIO + N0 group was significantly higher than that of the CK + N0 group (*p* < 0.05) (Supplementary Figure S1).

Regardless of HNFB inoculation, excessive nitrogen application had a negative impact on MBN and MBC in the rhizosphere soil, further exacerbating salt stress. N1 application significantly increased the MBC and MBP content in the rhizosphere soil, while reducing the degree of salt stress. Inoculation with HNFB also significantly increased MBN, MBC, and MBP levels in the rhizosphere soil under both N0 and N1 conditions, leading to a reduction in salt stress. Under excessive nitrogen application, HNFB significantly increased MBC and MBP in the rhizosphere soil. Under low-nitrogen conditions, HNFB significantly increased the total nitrogen content in the rhizosphere soil. The results of the two-way ANOVA further confirmed that the nitrogen application level and bacterial treatment had significant effects on the physical and chemical properties of the soil and showed interaction effects on the MBP, MBC, and EC (Table 1).

**Table 1:** Interaction analysis of nitrogen application levels and HNFB treatment.

Factor	MBC		MBN		EC		MBP		pH		Soil N	
	*F*	*p*	*F*	*p*	*F*	*p*	*F*	*p*	*F*	*p*	*F*	*p*
BIO	1218.518	0	32.215	0	420.963	0	149.756	0	0.011	0.918	4.379	0.058
N	952.109	0	15.569	0	70.994	0	22.995	0	10.626	0.002	8578.062	0
BIO*N	62.264	0	0.131	0.879	5.299	0.022	18.729	0	1	0.397	0.061	0.941

*F* and *p* values represent the results of the interaction analysis between nitrogen application levels and bacterial treatment (two-way ANOVA). HNFB, halotolerant nitrogen-fixing bacteria; MBN, microbial biomass nitrogen; MBC, microbial biomass carbon; MBP, microbial biomass phosphorus; EC, electrical conductivity; N, nitrogen; BIO, treatment wherein the three nitrogen application levels were set up with bacterial inoculation (*Bacillus subtilis* HG-15 + *Bacillus velezensis* JC-K3).

### 3.2 Effects of nitrogen fertilizer and exogenous HNFB application on AMF community composition in the apple rhizosphere

**Figure 1:**
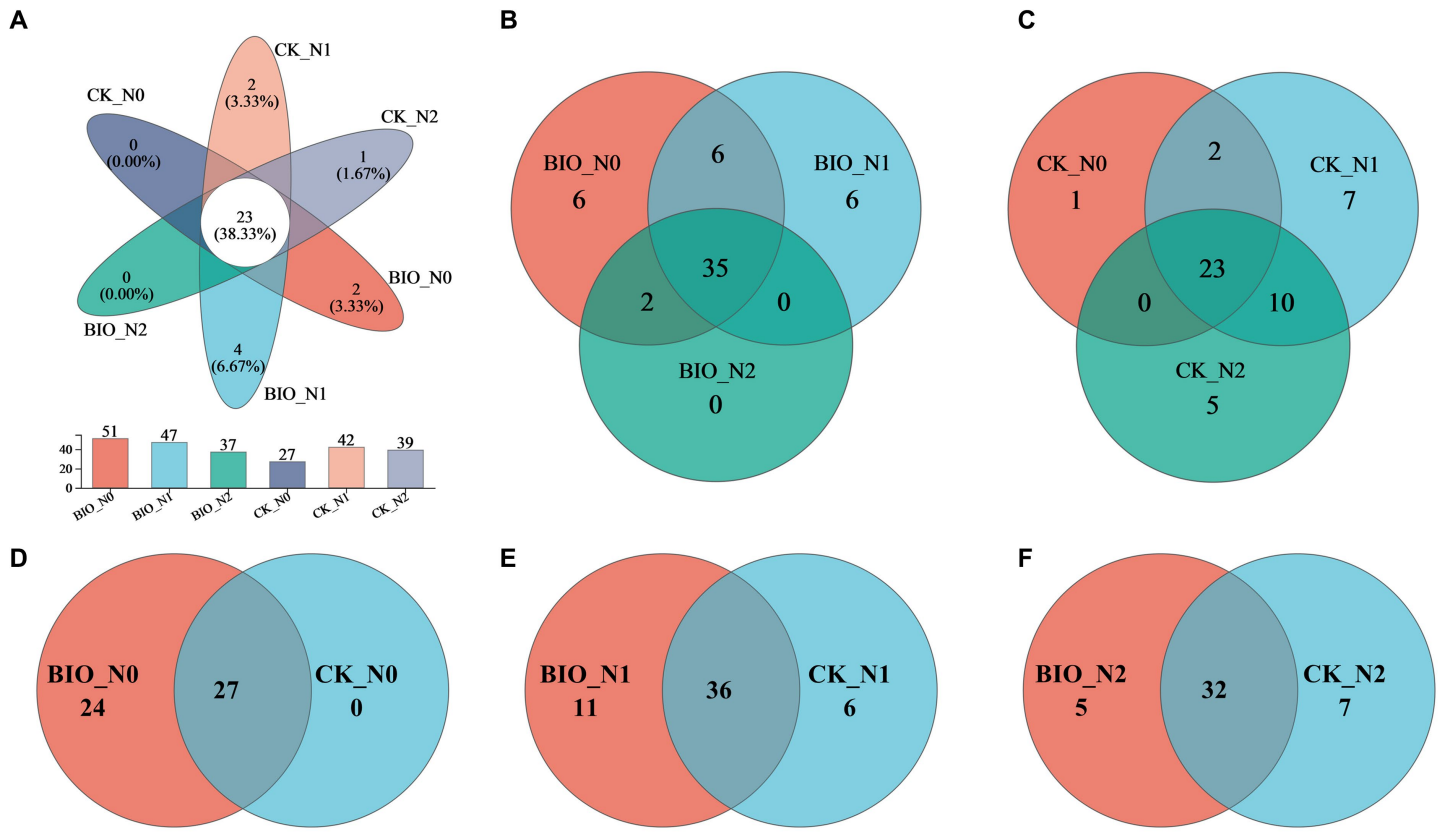
Venn diagrams depicting the AMF community structure in the different treatment samples at the OTU level. OTU, operational taxonomic unit. AMF, ascomycetous fungi; CK, control wherein the three nitrogen application levels were set up without bacteria; BIO, treatment wherein the three nitrogen application levels were set up with bacterial inoculation (*Bacillus subtilis* HG-15 + *Bacillus velezensis* JC-K3); N0, low nitrogen level; N1, normal nitrogen level; N2, high nitrogen level.

In total, 365,930 valid AMF sequences (average length, 215 bp) were obtained, accounting for 98.86% of the original sequences and covering most of the AMF community in the rhizosphere soil (Supplementary Figure S2). Based on a 97% similarity analysis, 60 OTUs were classified from the effective AMF sequences, including one phylum, three families, three genera, and 20 species. There were 23 common OTUs among the six treatments, 35 common OTUs in the BIO groups, and 23 common OTUs in the CK groups. The results showed that HNFB increased 12 OTUs in the apple rhizosphere (Figure 1). Therefore, excessive nitrogen application was not instrumental in increasing AMF species in the rhizosphere soil and HNFB inoculation enriched the AMF species in the rhizosphere soil under N0 and N1 levels.

Inoculation with exogenous HNFB increased the rhizosphere AMF community richness (Simpson, ACE and Chao index, Table 2) under N0 conditions and maintained rhizosphere AMF community richness under N1 and N2 conditions. After inoculation with exogenous HNFB, excessive nitrogen application significantly reduced AMF richness and diversity compared to those at low or normal nitrogen levels (Table 2). Two-way analysis further confirmed that nitrogen fertilizer and HNFB application significantly affected the AMF richness of the rhizosphere soil, and there was an interaction with the AMF richness. However, nitrogen fertilizer and HNFB had no significant effect on or interaction with the AMF diversity (Supplementary Table S1). The results of qRT-PCR revealed a gradual decline in the abundance of nitrogen-fixing bacteria in soil with an increase in nitrogen application rate. In addition, the abundance of AMF was significantly lower at N0 and N2 than at N1 (*p* < 0.05), and under the same level of nitrogen application, the abundances of nitrogen-fixing bacteria and AMF in the inoculated HNFB group were significantly higher than those in the CK group (*p* < 0.05) (Supplementary Figure S3).

**Table 2:** Diversity index of AMF in the apple rhizosphere soil samples from different treatment groups.

Sample	Sobs	Chao	Ace	Shannon	Simpson
BION0	38.00 ± 13.00	41.83 ± 7.65	51.82 ± 7.36	2.37 ± 0.36	0.15 ± 0.04
BION1	36.00 ± 3.00	36.61 ± 3.76	38.27 ± 5.09	2.21 ± 0.12	0.17 ± 0.02
BION2	30.33 ± 1.53	31.78 ± 1.58	31.95 ± 1.48	1.96 ± 0.04	0.23 ± 0.02
CKN0	24.00 ± 0.00	24.61 ± 0.35	28.5 ± 5.09	1.98 ± 0.02	0.19 ± 0.00
CKN1	34.00 ± 4.58	38.50 ± 9.76	42.45 ± 16.86	2.12 ± 0.12	0.17 ± 0.02
CKN2	27.33 ± 2.89	28.33 ± 4.04	28.52 ± 4.14	2.00 ± 0.18	0.18 ± 0.05
N treatment	0.277	0.105	0.091	0.141	0.078
Microbial treatment	0.042	0.035	0.077	0.120	0.940

N treatment indicate significant differences between the nitrogen application level groups for the same treatment (one-way ANOVA, *p* < 0.05). Microbial treatment indicate significant differences between the results of the different treatments for the same nitrogen application level (two-sided *t*-test). AMF, ascomycetous fungi; CK, control wherein the three nitrogen application levels were set up without bacteria; BIO, treatment wherein the three nitrogen application levels were set up with bacterial inoculation (*Bacillus subtilis* HG-15 + *Bacillus velezensis* JC-K3); N0, low nitrogen level; N1, normal nitrogen level; N2, high nitrogen level.

**Figure 2:**
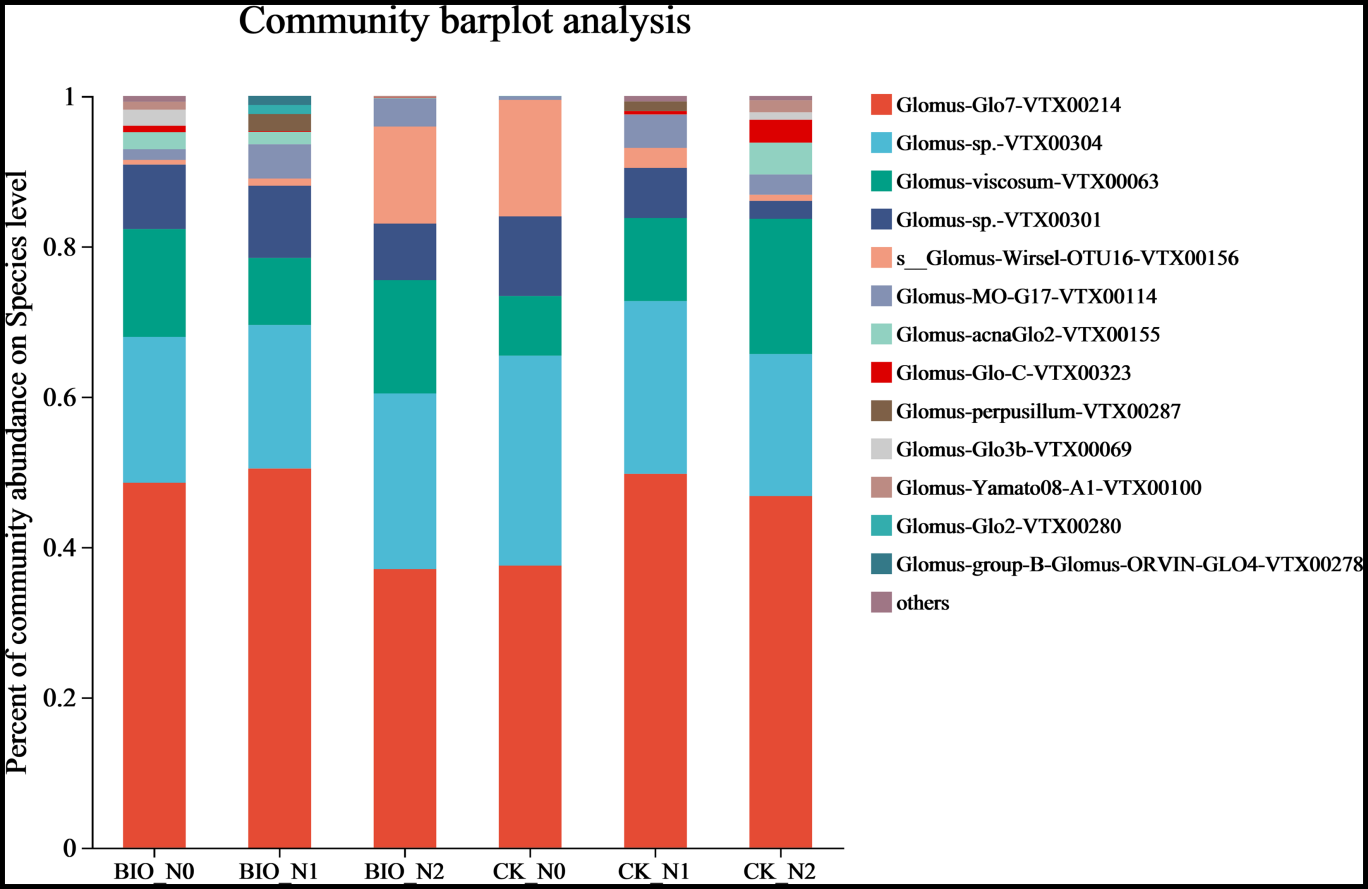
The relative abundance of AMF in the apple rhizosphere soil across the different treatment groups at the species level. AMF, ascomycetous fungi; CK, control wherein the three nitrogen application levels were set up without bacteria; BIO, treatment wherein the three nitrogen application levels were set up with bacterial inoculation (*Bacillus subtilis* HG-15 + *Bacillus velezensis* JC-K3); N0, low nitrogen level; N1, normal nitrogen level; N2, high nitrogen level.

**Figure 3:**
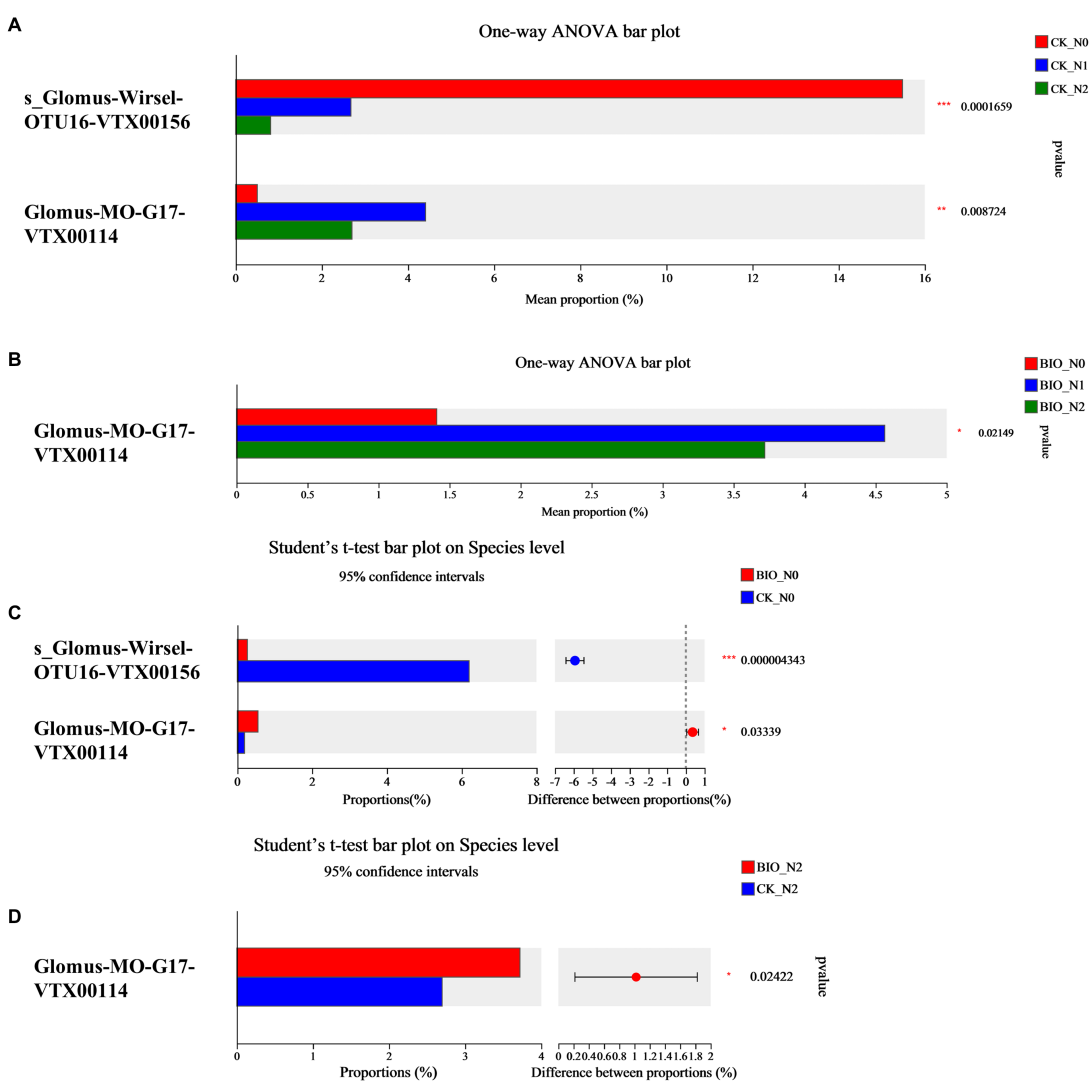
The significant differences in the relative abundance of AMF in the apple rhizosphere soil from different treatment groups at the species level. AMF, ascomycetous fungi; CK, control wherein the three nitrogen application levels were set up without bacteria; BIO, treatment wherein the three nitrogen application levels were set up with bacterial inoculation (*Bacillus subtilis* HG-15 + *Bacillus velezensis* JC-K3); N0, low nitrogen level; N1, normal nitrogen level; N2, high nitrogen level.

The top 10 most abundant AMF are shown in Figure 2. We found that the main AMF genus was *Glomus*. In detail, *Glomus-Glo7-VTX00214*, *Glomus-*sp.*-VTX00304*, *Glomus-viscosum-VTX00063*, and *Glomus-*sp.*VTX00301* were the dominant species in each treatment group (Figure 2; Supplementary Figure S4). Among the CK groups, the relative abundance of *s__Glomus-Wirsel-OTU16-VTX00156* in the N0 group was 15.48%, which was significantly higher than that in the N1 (2.68%) and N2 (0.81%) groups (*p* < 0.001) (Figure 3A; Supplementary Figures S4B,D,F). Among the BIO groups, the relative abundance of *Glomus-MO-G17-VTX00114* was 4.56% in the N1 treatment group, which was significantly higher than that in the N2 (3.72%) and N0 (1.41%) groups (*p* < 0.05) (Figure 3B; Supplementary Figures S4A,C,E). The relative abundance of *s__Glomus-Wirsel-OTU16-VTX00156* in the BIO group increased with increasing levels of applied nitrogen. Furthermore, the relative abundance of this fungus in the BIO + N0 group was significantly lower than that in the CK groups (Figure 3C) (*p* < 0.001). For the N0 and N2 treatment groups, the relative abundance of *Glomus-MO-G17-VTX00114* in the BIO group was significantly higher than that in the CK group (Figures 3C,D) (*p* < 0.05). The relative abundance of *Glomus-MO-G17-VTX00114* decreased after excess nitrogen application with or without exogenous HNFB inoculation (Figures 3A,B) (*p* < 0.05).

### 3.3 Effects of nitrogen fertilizer and exogenous HNFB application on AMF community function in the apple rhizosphere

**Figure 4:**
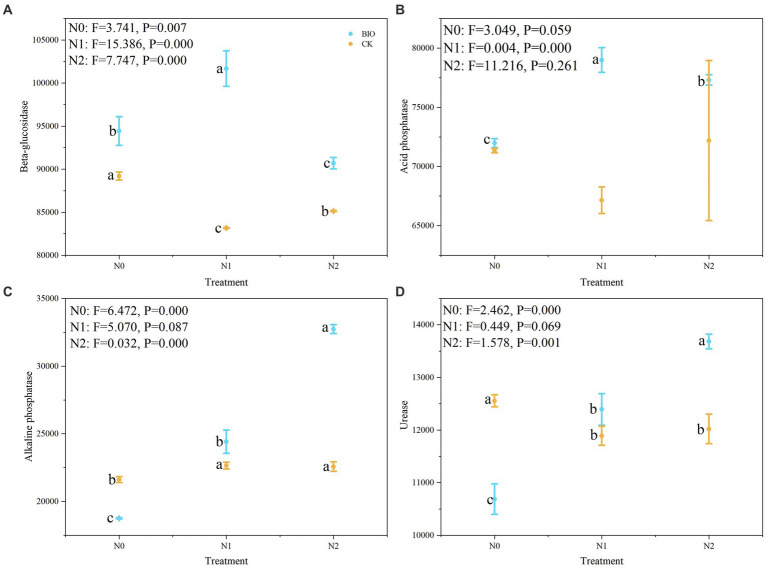
Relative levels of enzymes produced by the microbial community in the different treatment groups. The relative levels of **(A)** beta-glucosidase, **(B)** acid phosphatase, **(C)** alkaline phosphatase, and **(D)** urease in the rhizosphere after different treatments were detected using PICRUSt2 software. AMF, ascomycetous fungi; CK, control wherein the three nitrogen application levels were set up without bacteria; BIO, treatment wherein the three nitrogen application levels were set up with bacterial inoculation (*Bacillus subtilis* HG-15 + *Bacillus velezensis* JC-K3); N0, low nitrogen level; N1, normal nitrogen level; N2, high nitrogen level.

The PICRUSt2 software was used to predict the function of the microbial community detected in the samples based on the amplicon sequencing results, and the enzyme types produced by the flora in the different treatment groups and their relative levels were determined (Supplementary Table S2). The relative abundances of beta-glucosidase and acid phosphatase in the BIO + N1 group were significantly higher than those in the BIO + N0 and BIO + N2 groups (Figure 4A) (*p* < 0.05). The relative abundance of beta-glucosidase in the CK + N1 group was significantly lower than that in the CK N0 and N2 groups (Figure 4A) (*p* < 0.05). Acid phosphatase levels in the BIO group first increased and then decreased with increasing nitrogen application levels, and the BIO + N1 group had significantly higher levels than those in the BIO + N2 and BIO + N0 groups (*p* < 0.05). Nitrogen application in the CK group had no significant effect on the relative abundance of acid phosphatases (Figure 4B).

In contrast, in the BIO group, the relative abundances of alkaline phosphatase and urease increased with increasing nitrogen levels. The N2 group had significantly higher levels compared to the N0 and N1 groups (Figure 4C) (*p* < 0.05). There was no significant difference in the relative abundances of alkaline phosphatase and urease between the CK + N1 and CK + N2 groups. However, they were significantly higher than those in the CK + N0 group (Figures 4C,D) (*p* < 0.05). Urease levels in the BIO + N0 group were significantly higher compared to the BIO + N1 and N2 groups (*p* < 0.05), whereas urease levels in the CK + N0 group were significantly lower compared to the CK + N1 and N2 groups (Figure 4D) (*p* < 0.05). In conclusion, excessive nitrogen application reduced the relative abundance of beta-glucosidase, acid phosphatase, and urease, regardless of whether HNFB was inoculated. However, when HNFB was inoculated, the relative abundance of alkaline phosphatase significantly increased with increasing nitrogen application levels.

Under the same nitrogen application level, the relative abundance of beta-glucosidase in the BIO group was significantly higher than that in the CK group (*p* < 0.05) (Figure 4A). The relative abundance of acid phosphatase in the BIO + N1 group was significantly higher than that in the CK + N1 group (*p* < 0.05); however, there was no significant difference due to HNFB inoculation at the N0 and N2 levels (Figure 4B). The relative abundances of alkaline phosphatase and urease in the CK + N0 and N2 groups were significantly higher than those in the BIO + N0 and N2 groups (*p* < 0.05), and there were no significant differences between the CK + N1 and BIO + N1 groups (Figures 4C,D).

Therefore, inoculation with exogenous HNFB significantly increased the relative abundance of beta-glucosidase at the N0 level but significantly reduced the abundance of alkaline phosphatase and urease. The relative abundances of beta-glucosidase and acid phosphatase significantly increased at the N1 level, and the relative abundances of beta-glucosidase, alkaline phosphatase, and urease significantly increased at the N2 level.

### 3.4 Correlation analysis of environmental factors

We conducted an analysis of the variance inflation factor (VIF). The VIF values of the environmental factors MBP (VIF = 4.59), MBN (VIF = 5.90), pH (VIF = 1.48), EC (VIF = 7.70), and soil N (VIF = 1.53) were less than 10 and could be used for RDA. However, the VIF of MBC was 19.63, which was greater than 10, indicating collinearity with other environmental factors. Therefore, MBC was removed from the RDA to ensure an accurate assessment of the effect of soil physical and chemical factors on structural latitude biodiversity.

**Figure 5:**
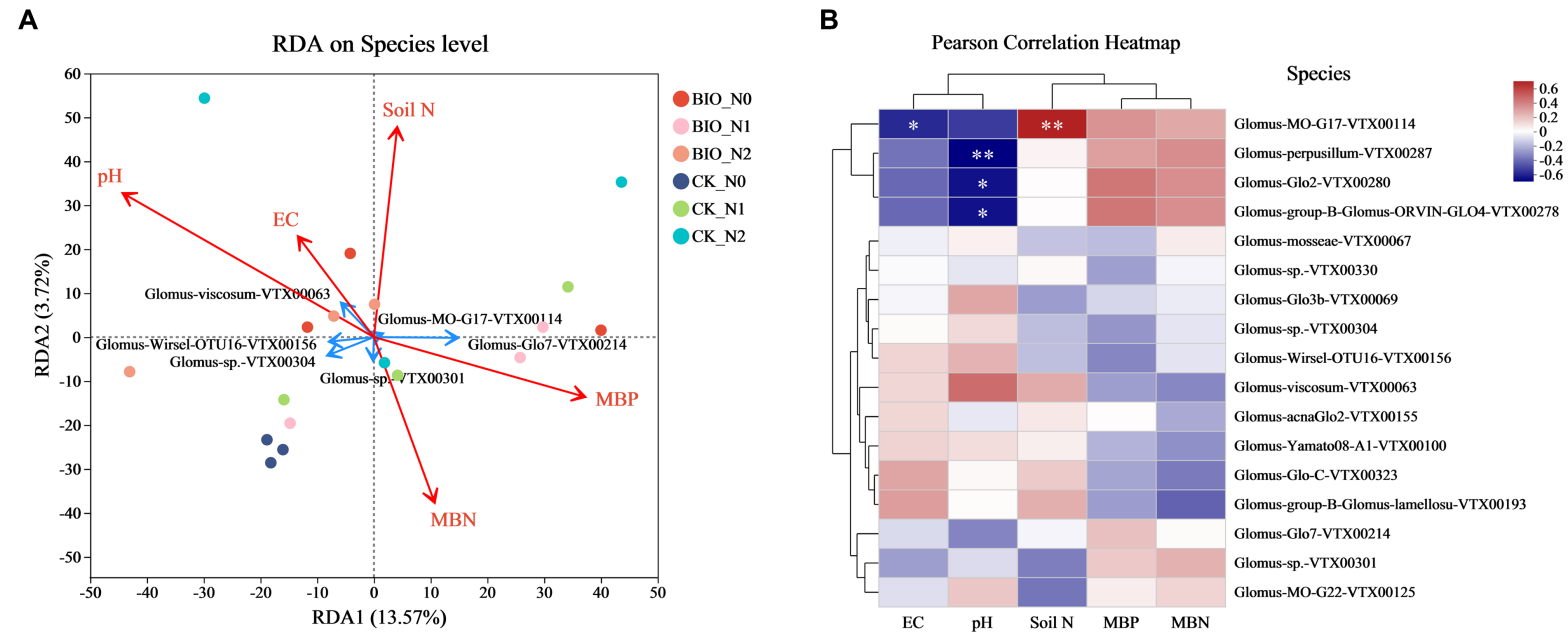
**(A)** Redundancy analysis of the AMF community based on Bray-Curtis distances. Different colored points represent samples from the different treatments. The closer the points of the two samples are, the more similar their species compositions. The red arrows indicate environmental factors, and the blue arrows indicate the dominant species. **(B)** Correlation between different environmental factors and AMF species. The X-axis and Y-axis represent environmental factors and species, respectively, and the right legend shows the color interval of the different R values. The left and upper sides represent the clustering trees for the species and environmental factors, respectively. *p ≤ 0.05, **p ≤ 0.01, ***p ≤ 0.001. This analysis is based on a weighted UniFrac matrix. AMF, ascomycetous fungi; CK, control wherein the three nitrogen application levels were set up without bacteria; BIO, treatment wherein the three nitrogen application levels were set up with bacterial inoculation (*Bacillus subtilis* HG-15 + *Bacillus velezensis* JC-K3); N0, low nitrogen level; N1, normal nitrogen level; N2, high nitrogen level.

Significant differences in microbial structure were observed after different nitrogen treatments and the addition of HNFB. By combining environmental factor analysis, we found that EC and pH were positively correlated with *Glomus-viscosum-VTX00063* and *s__Glomus-Wirsel-OTU16-VTX00156*. Soil N and MBP were positively correlated with *Glomus-MO-G17-VTX00114*, while MBN was positively correlated with *Glomus-*sp.*VTX00304* cells (Figure 5A). The results of the correlation analysis of the environmental factors were consistent with those of the RDA. The clustering relationship between EC and pH was similar, with EC being significantly negatively correlated with *Glomus-MO-G17-VTX00114* (*p* < 0.05). The pH showed a significant negative correlation with *Glomus-perpusillum-VTX00287* (*p* < 0.01), *Glomus-Glo2-VTX00280* (*p* < 0.05), and *Glomus-group-B-Glomus-ORVIN-GLO4-VTX00278* (*p* < 0.05) levels. Soil N was significantly positively correlated with *Glomus-MO-G17-VTX00114* (*p* < 0.01) (Figure 5B).

**Figure 6:**
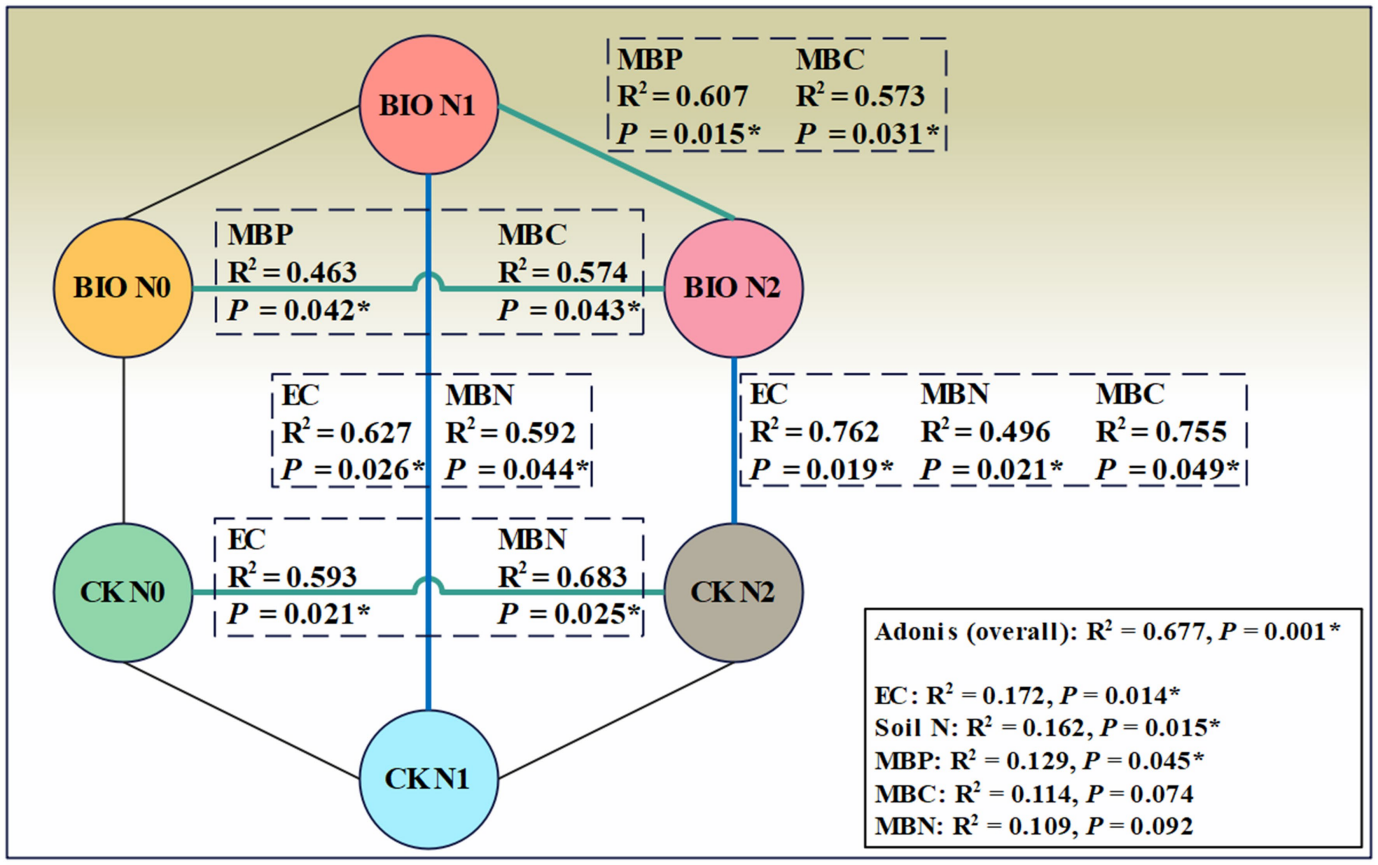
PERMANOVA analysis of the AMF community in the six treatment groups. AMF, ascomycetous fungi; CK, control wherein the three nitrogen application levels were set up without bacteria; BIO, treatment wherein the three nitrogen application levels were set up with bacterial inoculation (*Bacillus subtilis* HG-15 + *Bacillus velezensis* JC-K3); N0, low nitrogen level; N1, normal nitrogen level; N2, high nitrogen level.

PERMANOVA was used to interpret the correlation between different environmental factors and the AMF community structure in various samples. A permutation test was employed to determine the statistical significance of the division. The results revealed that EC, soil N, and MBP significantly influenced the samples (R^2^ = 0.677, *p* = 0.001) (Figure 6). Both the BIO + N0 and BIO + N2 groups, as well as the BIO + N1 and BIO + N2 groups, were significantly impacted by MBP (*p* < 0.05) and MBC (*p* < 0.05). Similarly, the CK + N0 and CK + N2 groups were significantly affected by EC (*p* < 0.05) and MBN (*p* < 0.05). Similarly, the BIO + N1 and CK + N1 groups were significantly affected by EC (*p* < 0.05) and MBN (*p* < 0.05). Additionally, the BIO + N2 and CK + N2 groups were significantly affected by EC (*p* < 0.05), MBN (*p* < 0.05), and MBC (*p* < 0.05). The pH did not have an overall significant effect, nor did it differ significantly between groups. Therefore, nitrogen treatments primarily influenced the AMF community structure in the rhizosphere soil by regulating the content of MBP and MBC, following inoculation with exogenous HNFB. In the absence of inoculation with exogenous HNFB, nitrogen primarily affected the AMF community structure by regulating the EC and MBN content in the rhizosphere soil. Under the N1 and N2 treatments, significant differences in the AMF community structure between the BIO and CK groups were mainly attributed to differences in EC and MBN.

**Figure 7:**
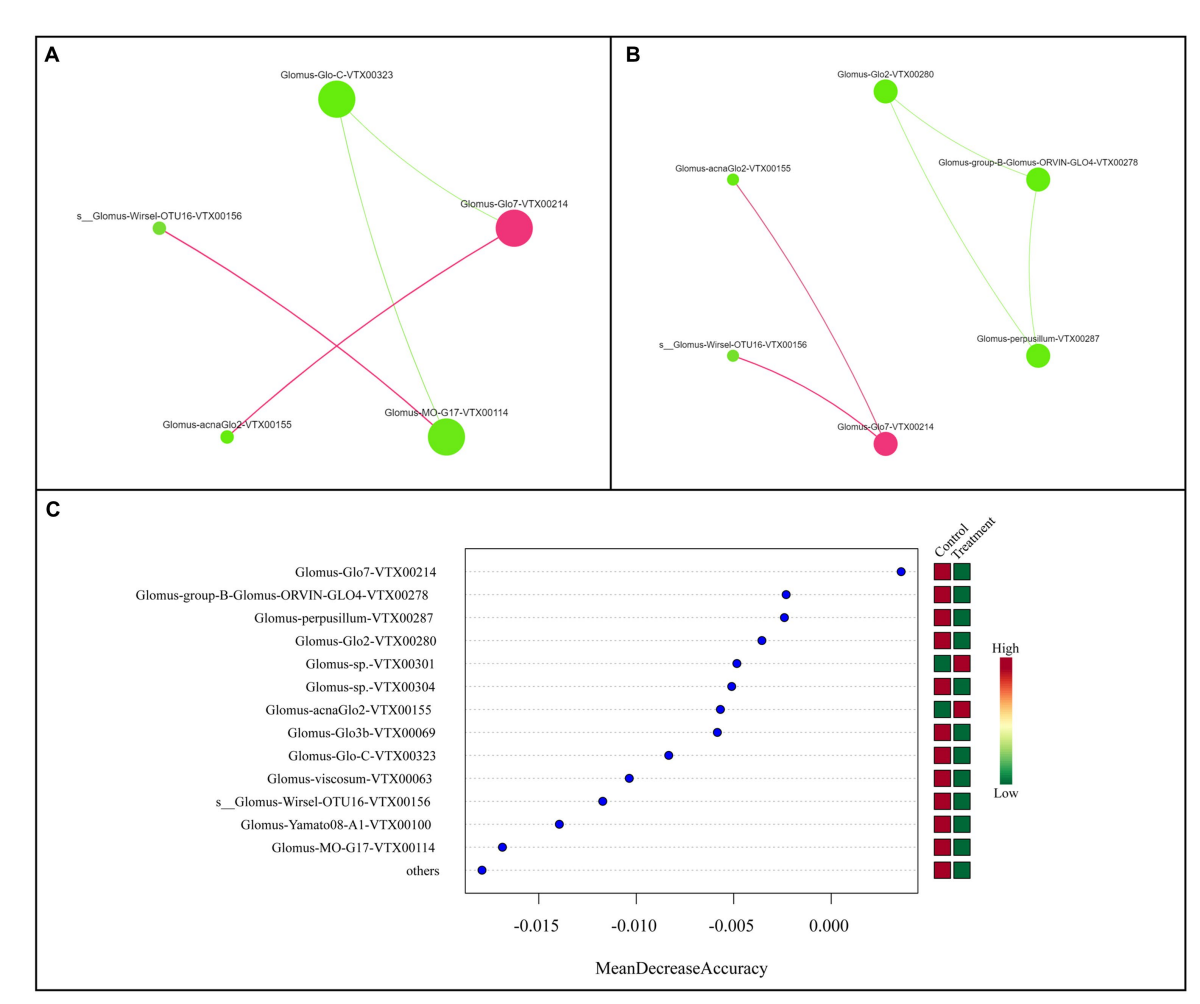
Colinear network analysis and random forest model showing the relationship between key AMF species. Collinear network analysis of the **(A)** CK and **(B)** BIO groups. **(C)** Random forest model, wherein the ordinate is the species and the abscissa is the measured value of species importance. AMF, ascomycetous fungi; CK, control wherein the three nitrogen application levels were set up without bacteria; BIO, treatment wherein the three nitrogen application levels were set up with bacterial inoculation (*Bacillus subtilis* HG-15 + *Bacillus velezensis* JC-K3); N0, low nitrogen level; N1, normal nitrogen level; N2, high nitrogen level.

This study examined the collinear model of AMF related to nitrogen fertilizer and HNFB application by constructing two molecular ecological networks. In the AMF co-occurrence network of the CK and BIO samples, *Glomus-Glo7-VTX00214* played a vital role (Figures 7A,B). Consistent with the results of the flora composition depicted in Figure 2, *Glomus-Glo7-VTX00214* had the highest relative abundance in each treatment, and its relative abundance in the BIO group was greater than that in the CK group under the N0 and N1 treatments. Furthermore, the results from the random forest analysis further demonstrated that *Glomus-Glo7-VTX00214* was the most crucial predictor of the AMF structure in the apple rhizosphere soil (Figure 7C).

## 4 Discussion

### 4.1 Differences between the effects of nitrogen application levels and that of exogenous HNFB inoculum on soil physical and chemical properties

In our results, nitrogen application led to significant changes in soil MBC and MBN content. Most notably, excessive N application reduced MBC and MBN regardless of whether HNFB was inoculated (Supplementary Figures S1A,B). The main reason underling this change may be that excessive nitrogen can increase nitrogen uptake by crops, but it also alters soil nitrogen effectiveness (Cairney, 2011), affecting nitrogen absorption and reabsorption by plants (Ostonen et al., 2011) and the exchange of metabolites between roots and microorganisms. The competition between plants and microorganisms for available nutrients, such as MBC and MBN, under salt stress was intensified, thereby reducing the available MBC and MBN (Ganeshamurthy and Reddy, 2015). Furthermore, exogenous HNFB significantly increased microbial biomass in rhizosphere soil under both low and normal nitrogen conditions and reduced salt stress. We speculate the main reasons for the above finding is that inoculation with PGPR can increase the number and diversity of rhizosphere microorganisms, improve microbial nitrogen fixation, promote metabolite exchange, and enhance ion-transport efficiency between roots and rhizosphere microorganisms, thereby increasing nutrient-use efficiency (Zhang et al., 2022).

We found that after HNFB inoculation, although all soil MBC/MBN ratios were > 6, these ratios decreased with increasing nitrogen application levels (Supplementary Figure S1). On one hand, this indicates that fungi still play a beneficial role in the rhizosphere soil, ensuring relatively stable soil carbon sequestration capacity (Chowdhury et al., 2011; Morrissey et al., 2013). On the other hand, excessive nitrogen application further reduces the MBC/MBN ratio and leads to a shift in the apple rhizosphere soil flora from fungi to bacteria. However, in the control group without HNFB inoculation, the soil MBC/MBN ratio increased with increasing nitrogen application levels (Supplementary Figure S1). Therefore, this finding suggests that an improved HNFB composition might increase the presence of synergistic fungal species and mitigate the adverse effects of the soil microbial biomass on the C/N imbalance caused by a purely bacterial flora.

Consistent with previously reported findings (Silva et al., 2013; Dynarski and Houlton, 2017), in our results, we observed a decrease in the abundance of nitrogen-fixing bacteria in response to increased nitrogen application rates (Supplementary Figure S3A). The main reason underlying this observation may be that nitrogen levels are negatively correlated with the number of nitrogen-fixing bacteria and nitrogen-fixing activity. Lower levels of nitrogen application have been shown to be more conducive to the nitrogen-fixing activity of nitrogen-fixing microorganisms (Sarathchandra et al., 1988; Bailey et al., 2002; Bagyaraj, 2010). These findings indicate that the nitrogen application rate can be controlled within a reasonable range to ensure a high amount and activity of microbial nitrogen fixation in saline-alkali soil.

Phosphorus is a limiting factor that affects the activity of nitrogen-fixing microorganisms. However, both low and excessive nitrogen application could not assist microbial agents in increasing MBP, which significantly increased (*p* < 0.05) under normal nitrogen application levels (Supplementary Figure S1C). Under the same nitrogen application conditions, MBP in the CK group was significantly lower than that in the BIO group, which further confirms that HNFB plays a beneficial role in improving soil microbial vigor and nutrient content when applied to saline soil (Primieri et al., 2021; Liu et al., 2022). The interaction between nitrogen application and exogenous HNFB on MBP further confirms the critical role of the balance between nitrogen and phosphorus in maintaining soil microbial activity (Table 1). qRT-PCR results revealed that the abundance of arbuscular fungi was significantly higher under N1 conditions than under N0 and N2 conditions and was higher under N2 conditions than under N0 (Supplementary Figure S3B). The same trend was observed for MBP treated with HFNB inoculation. Although it is unclear how native AMF communities respond to diminishing soil phosphorus effectiveness, AMF can obtain phosphorus via exoenzymes (Meeds et al., 2021). Therefore, the effects of nitrogen application and exogenous HNFB on AMF community structure are also important factors influencing phosphorus accumulation in the microbial biomass.

Generally, when there is a high input of nitrogen, it can enhance soil nitrification, which leads to an increase in the release of H^+^ during nitrogen transformation and soil acidification (Wang et al., 2023). However, our results showed that the rhizosphere soil pH in the BIO + N1 group was significantly lower than that of the BIO + N0 and BIO + N2 groups. On the other hand, there were no significant differences among the other groups and treatments (Supplementary Figure S1E). Therefore, applying excessive amounts of urea in saline soils may not promote nitrification in the soil, and it is difficult to significantly affect soil pH by simply adjusting the amount of nitrogen applied. In contrast, when HNFB was inoculated under normal nitrogen application, it reduced the soil pH. This suggests that normal nitrogen application helps maintain a higher HNFB-plant biochemical response.

### 4.2 Differences in the effects of nitrogen application and that of HNFB inoculum on the AMF community

In nitrogen-deficient ecosystems, plants increase their nitrogen use efficiency (NUE) to meet growth needs (Li et al., 2016). However, excessive nitrogen application inhibits the symbiotic relationship between plants and microorganisms (Laws and Graves, 2005), including AMF (Panneerselvam et al., 2023). The effects of nitrogen treatment and HNFB on AMF community composition and abundance during short-term fertilization can help explain changes in soil physicochemical properties and enzyme activities. Low nitrogen treatment, due to the lack of carbon return from the host, reduces fungal colonization in plants, particularly AMF (Irving et al., 2022). This may be why N1 had the highest AMF abundance and N0 had the lowest abundance in the CK group (Table 2). In contrast, the AMF community richness and diversity in the BIO group were higher than those in the CK group at the same nitrogen application level, suggesting a more sustainable low-input agricultural cropping system, consistent with a previous study (Primieri et al., 2021).

In the BIO group, there was no significant difference in AMF diversity between the treatment without nitrogen application and the N1 treatment. However, the diversity in the N0 group was significantly higher than that in the N2 group (Table 2). The negative effect of excess nitrogen on the rhizosphere AMF community in the apple rhizosphere aligns with the findings of previous studies (Zeng et al., 2021). Hence, there is compelling evidence to suggest that long-term and/or increased application of exogenous HNFB under salt stress conditions can effectively improve crop rhizosphere soil microbial ecology, positively influencing AMF community structure and diversity. The bacterial members of exogenous HNFB are indispensable for maintaining functional stability.

Similarly, nitrogen and exogenous HNFB caused changes in the AMF community composition (Figures 2, 5). The dominant relative abundance of *s__Glomus-Wirsel-OTU16-VTX00156* in the CK + N0 group decreased significantly with increased nitrogen content and HNFB inoculation (Figure 2; Supplementary Figure S4). This suggests that this strain is better adapted to low-nitrogen conditions. It is likely that exogenous HNFB and this strain have a mutually inhibitory relationship, and the combination of HNFB and this strain may reduce the effect of bacterial action.

### 4.3 Effects of nitrogen application and exogenous HNFB inoculum on AMF community function

Previous studies have found that a sufficient nitrogen source is conducive to the synthesis of phosphatases (Braun et al., 2010), particularly alkaline phosphatases (Chen et al., 2023b). Our results demonstrate that the activities of beta-glucosidase, alkaline phosphatase, acid phosphatase, and urease were significantly affected by the nitrogen application level and exogenous HNFB (Figure 6). Nitrogen fertilizer can increase soil β-glucosidase and alkaline phosphatase activities while reducing urease activity. This contradicts the results of the β-glucosidase and urease assessments conducted in this study (Figure 4). One possible reason is that the number, diversity, and richness of microorganisms in salt-stressed soil are lower compared to those in regularly cultivated land (Chen et al., 2021). The microbial community structure in the rhizosphere soil is influenced by crop root metabolites and differs significantly from that in the topsoil (Wang et al., 2021). Under the N1 treatment, nitrogen-fixing bacteria expedite the conversion of soil substances, enhance metabolism in plant roots, promote shedding, and increase soil organic matter content. As a result, enzymatic reaction substrates and enzyme activity are elevated (George et al., 2006). An increase in alkaline phosphatase levels may be associated with an increase in MBC content as nitrogen application levels rise. MBC is a major predictor of the abundance of microorganisms carrying phoD and is positively correlated with alkaline phosphatase activity (Luo et al., 2019). The decrease in urease activity can be attributed to the inhibition of released urea (NH_4_^+^) hydrolysis products, as urease is involved in the nitrogen release process and NH_4_^+^ is considered an end product of urease. Microbial communities determine the relative abundance of enzyme-encoding genes and influence their expression. Therefore, significant differences in soil enzyme activity may be related to differences in microbial biomass and composition.

Exogenous HNFB, nitrogen application, indigenous flora, and plants are closely linked, and focusing solely on HNFB or nitrogen application may not provide a deeper understanding of the process of soil phosphorus transformation under real-world production conditions (Smith and Read, 2008). When soil phosphorus effectiveness is low, AMF can accelerate soil organic phosphorus mineralization and inorganic phosphorus activation by secreting phosphatases and organic acids (Fujii et al., 2012; Fan et al., 2019). For example, nitrogen application enhances acid phosphatase activity, increases the participation in ester-phosphate bond hydrolysis in soil organic phosphorus, and releases orthophosphate for plant uptake. Moreover, organic acids can release orthophosphate from the medium and other readily decomposable forms of soil inorganic phosphorus by chelating iron and aluminum (Lin et al., 2020). However, once AMF infects plant roots, if there is a change in the AMF community structure, the polyphosphate absorbed by the hyphae outside the roots is degraded into orthophosphate (Solaiman et al., 1999). This degradation promotes soil phosphorus transformation and plant phosphorus absorption.

### 4.4 The positive response of key AMF species to exogenous HNFB

The triple symbiotic system, consisting of nitrogen-fixing bacteria, AMF, and crops, synergistically promotes crop biomass and nitrogen fixation. This growth promotion effect is significantly superior to that of rhizobia, AMF, and crop symbionts (Primieri et al., 2021). Previous studies have shown that AMF contributes more to host growth under stressful conditions compared to normal conditions (Feng et al., 2020). Our results also confirm that AMF can establish ineffective interactions with exogenous HNFB and highlight the presence of core strains and auxiliary functions that have important synergistic effects. This synergy resembles that reported for other crops such as *Glycine max* (Wang et al., 2011), *Sibiraea angustata* (He et al., 2021), and *Amorpha canescens* (Larimer et al., 2016), and it may play a crucial role in sustainable low-input agricultural planting systems (Artursson et al., 2006). However, these effects depend on the specific combination of species involved in the interaction, specifically HNFB and/or AMF. A meta-analysis has even reported conflicting results, contradicting the hypothesis that AMF and nitrogen-fixing bacteria always exhibit synergistic effects in different plants (Larimer et al., 2010). This suggests that the occurrence of any synergistic effect may depend on the properties of the microsymbionts involved, as well as the spatial and temporal scales, the host, and/or the environmental conditions. The findings of our study provide a theoretical reference for the interaction between HNFB and AMF, with an emphasis on the dominance of *Bacillus*.

AMF can form a complex mycelial network with plants, affecting plant growth and stress resistance. *Glomus* is the dominant genus of the AMF of many soil types, and it was the only one genus was identified in this study. Of course, this result is not unique. For example, more than 740,000 valid sequences were obtained from 77 citrus root samples, 99% of which were of *Glomus*; furthermore, *Glomus-MO-G17-VTX00114* was confirmed to be a key species to ensure the stability of the AMF community in the rhizosphere and improve crop stress resistance (Song et al., 2015). Accordingly, our study further confirmed that the relative abundance of *Glomus-MO-G17-VTX00114* would be reduced by excessive nitrogen application (Figures 3A,B) and could be increased by HFNB application (Figures 3C,D). Moreover, *Glomus-Glo7-VTX00214* showed potential as an auxiliary HNFB strain in the apple rhizosphere AMF. Therefore, these results suggest an effective way to improve the relative abundance of key AMF species in the apple rhizosphere. Our proposed explanations are: (1) salt stress exerts a filtering effect on AMF (Rath et al., 2018), and *Glomus* has a significant advantage in terms of salinity tolerance or competitiveness; (2) plant variety is the most important factor affecting the composition of rhizosphere microorganisms (Edwards et al., 2023). Among AMF, *Glomus* may have an extremely close interaction with the root system of the examined apple plant, specifically selected for its variety. Simultaneously, several application studies have found that *Glomus* can be used to promote the growth of saline-alkali crops (Ouziad et al., 2006; Jahromi et al., 2008; Latef and He, 2014; Bharti et al., 2016; Amir and Samira, 2023) and indicate that the *Glomus* species has great potential for application in saline-alkali land agricultural production.

In this study, the relative abundance of *Glomus-MO-G17-VTX00114* for the N1 treatment in the BIO and CK groups was significantly higher than that in the N0 and N2 treatment groups. Generally, nitrogen fertilization is more beneficial to crops and the environment; this strain likely forms a synergistic relationship with the two nitrogen-fixing bacteria that were inoculated together to improve the rhizosphere AMF community structure and salt tolerance in apple trees in saline land (Figure 3). *Glomus-Glo7-VTX00214* was confirmed to play an important role in the AMF community composition and was the most important predictor (Figure 6). This strain can be used as an auxiliary strain to improve the structural and functional stability of the core flora. Here, nitrogen application was performed to increase the accuracy of the results. Moreover, the results of the flora composition and key species assessments were based on accurately identified and named dominant strains, ensuring that key strains could be isolated and cultured. Given the key role of AMF in plant productivity, nutrient cycling, and ecosystem responses to global change, a deeper understanding of the cross-scale coupling between plants and AMF diversity will reduce the uncertainty of ecosystem consequences when predicting species gains and losses (Fei et al., 2021). Therefore, the results of this study provide an important reference for the construction of a multitrophic synthetic HNFB with a more reasonable structure and greater diversity of species.

In general, our results showed that adding inorganic nitrogen alone weakened the function of AMF, whereas inoculation with HNFB stimulated its activity, thereby improving the salt and alkali resistance of plants. Excessive nitrogen application has a negative effect on the structure and function of the AMF community, which is worse than that under low nitrogen conditions. The core and auxiliary strains, determined through complex treatment and conditions in field experiments, are crucial references for the artificial synthesis of HNFB strains that have the ability to stably colonize saline-alkali land and exhibit a stronger synergistic effect. Thus, these results provide an overall “landscape” of the apple-AMF association in agricultural settings in major apple-production areas under different N application levels in China. They should serve as a guide for the future development of major apple-adapted AMF as potential bio-fertilizers.

## 5 Conclusion

This study revealed the uniqueness of the AMF community in the apple rhizosphere in saline-alkali soil and indicated that the AMF community was significantly affected by different N application levels and HNFB inoculation. This is evidenced by following findings. Inoculation with exogenous HNFB promoted soil nutrient accumulation and alleviated salt stress; simultaneously, exogenous HNFB increased the richness of rhizosphere AMF communities in low-nitrogen conditions and maintained their richness under both normal and high-nitrogen application conditions. However, excessive nitrogen application significantly reduced the richness and diversity of AMF after exogenous HNFB inoculation, highlighting the ecological risk associated with excessive nitrogen application in saline soil for the AMF community. Furthermore, in the apple rhizosphere AMF, the core strains *Glomus-MO-G17-VTX00114* and *Glomus-Glo7-VTX00214* exhibited potential as core and auxiliary HNFB strains, respectively. The new strain combination analyzed in this study is expected to be developed into a multitrophic HNFB with a more balanced structure and a greater variety of species. Considering the importance of AMF for crop growth in saline soil, it is essential to understand the response mechanisms of the apple and rhizosphere microorganisms to nitrogen application and exogenous HNFB for the sustainable management of apple agriculture in saline-alkali soil. Although this study did not accurately determine the appropriate amount of nitrogen fertilizer for apple tree growth in saline land, it provides data for exploring the relationship between exogenous HNFB-plant-AMF communities under different nitrogen application levels in complex habitats. This study could contribute to enhancing the salt tolerance of crops in saline land and reducing the reliance on chemical nitrogen fertilizers.

## Data availability statement

The datasets presented in this study can be found in online repositories. The names of the repository/repositories and accession number(s) can be found in the article/Supplementary material.

## Author contributions

CJ: Funding acquisition, Methodology, Writing – original draft, Writing – review & editing. YG: Conceptualization, Methodology, Writing – original draft. HZ: Data curation, Writing – original draft. YZ: Formal analysis, Software, Writing – original draft. ZX: Data curation, Investigation, Writing – original draft. JL: Funding acquisition, Software, Writing – original draft. JZ: Formal analysis, Software, Writing – original draft. ZL: Data curation, Writing – original draft. HC: Formal analysis, Funding acquisition, Writing – original draft. KL: Funding acquisition, Methodology, Project administration, Writing – original draft.

## Supplementary material

SUPPLEMENTARY FIGURE S1 Effects of different applied nitrogen levels and HNFB on the chemical properties of rhizosphere soil. The **(A)** MBN, **(B)** MBC, **(C)** MBP, **(D)** EC, **(E)** pH, and **(F)** soil N content in the different treatment groups is indicated. The F and *p* values represent significant differences in the results of different treatments within the same nitrogen application level (Student’s *t*-test); lowercase letters indicate significant differences in different nitrogen application levels for the same treatment (one-way ANOVA, *p* < 0.05). HNFB, halotolerant nitrogen-fixing bacteria; MBN, microbial biomass nitrogen; MBC, microbial biomass carbon; MBP, microbial biomass phosphorus; EC, electrical conductivity; N, nitrogen; CK, control wherein the three nitrogen application levels were set up without bacteria; BIO, treatment wherein the three nitrogen application levels were set up with bacterial inoculation (*Bacillus subtilis* HG-15 + *Bacillus velezensis* JC-K3); N0, low nitrogen level; N1, normal nitrogen level; N2, high nitrogen level.

SUPPLEMENTARY FIGURE S2 Rarefaction and Shannon curves of rhizosphere microorganisms in different treatment groups at the OTU level. OTU, operational taxonomic unit; CK, control wherein the three nitrogen application levels were set up without bacteria; BIO, treatment wherein the three nitrogen application levels were set up with bacterial inoculation (*Bacillus subtilis* HG-15 + *Bacillus velezensis* JC-K3); N0, low nitrogen level; N1, normal nitrogen level; N2, high nitrogen level.

SUPPLEMENTARY FIGURE S3 Abundances of nitrogen-fixing bacteria **(A)** and arbuscular mycorrhizal fungi **(B)** in the apple rhizosphere soil of different treatment groups.

SUPPLEMENTARY FIGURE S4 Relative abundance (percentage) of AMF species in the apple rhizosphere soil from different treatment groups.

SUPPLEMENTARY TABLE S1 Two-way ANOVA for assessing the effect of nitrogen fertilizer and HNFB on diversity indices of AMF communities.

SUPPLEMENTARY TABLE S2 Statistical analysis results of KEGG functional abundance in AMF communities.
