# Supplementary figures and images for: Interactions between halotolerant nitrogen-fixing bacteria and arbuscular mycorrhizal fungi under saline stress
Source: Front Microbiol. 2024 Mar 13;15:1288865. doi: 10.3389/fmicb.2024.1288865 (PMC11022851; doi:10.3389/fmicb.2024.1288865)

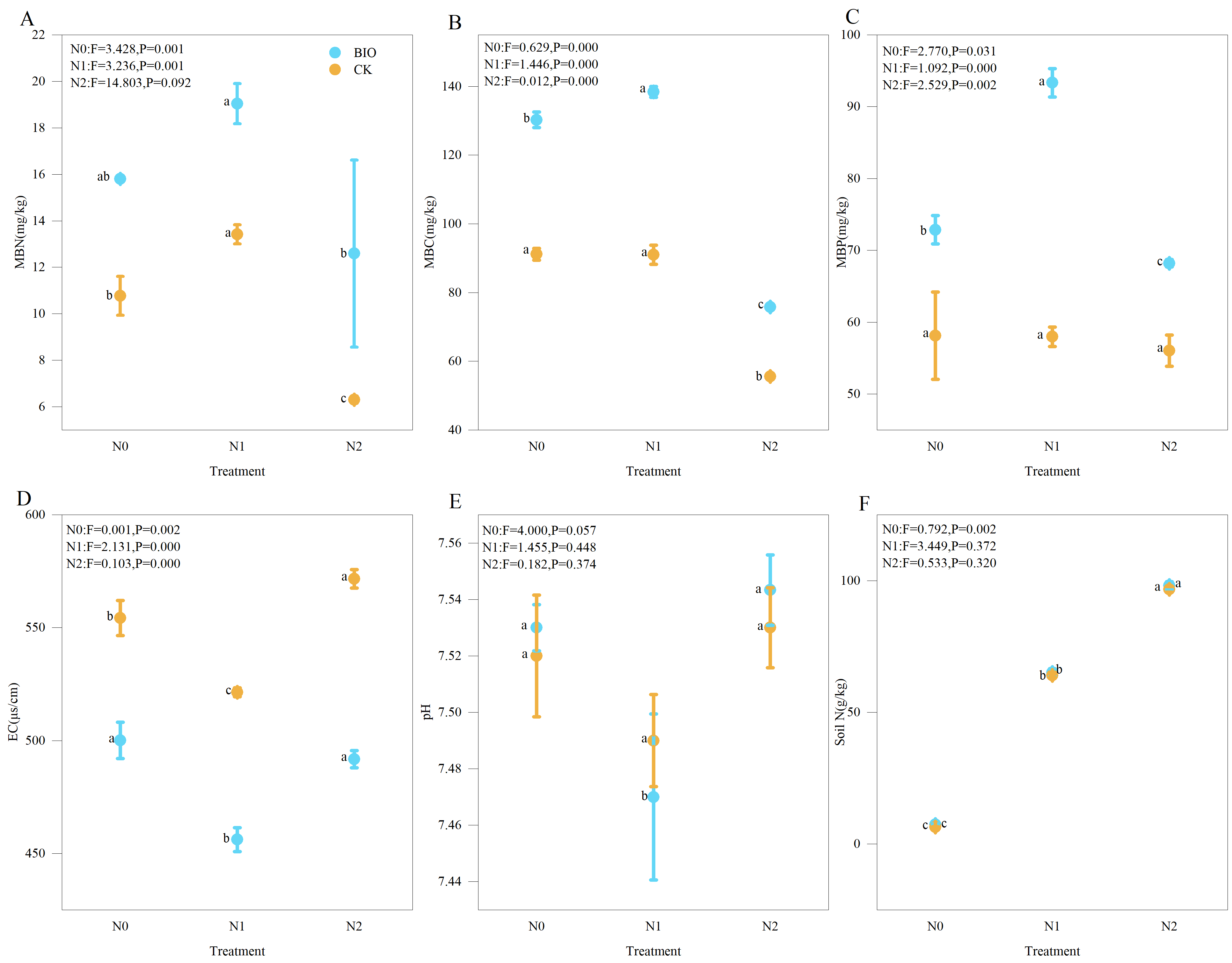

Supplement: SUPPLEMENTARY FIGURE S1 — Effects of different applied nitrogen levels and HNFB on the chemical properties of rhizosphere soil. The (A) MBN, (B) MBC, (C) MBP, (D) EC, (E) pH, and (F) soil N content in the different treatment groups is indicated. The F and p values represent significant differences in the results of different treatments within the same nitrogen application level (Student’s t-test); lowercase letters indicate significant differences in different nitrogen application levels for the same treatment (one-way ANOVA, p < 0.05). HNFB, halotolerant nitrogen-fixing bacteria; MBN, microbial biomass nitrogen; MBC, microbial biomass carbon; MBP, microbial biomass phosphorus; EC, electrical conductivity; N, nitrogen; CK, control wherein the three nitrogen application levels were set up without bacteria; BIO, treatment wherein the three nitrogen application levels were set up with bacterial inoculation (Bacillus subtilis HG-15 + Bacillus velezensis JC-K3); N0, low nitrogen level; N1, normal nitrogen level; N2, high nitrogen level. [file Image_1.tif]

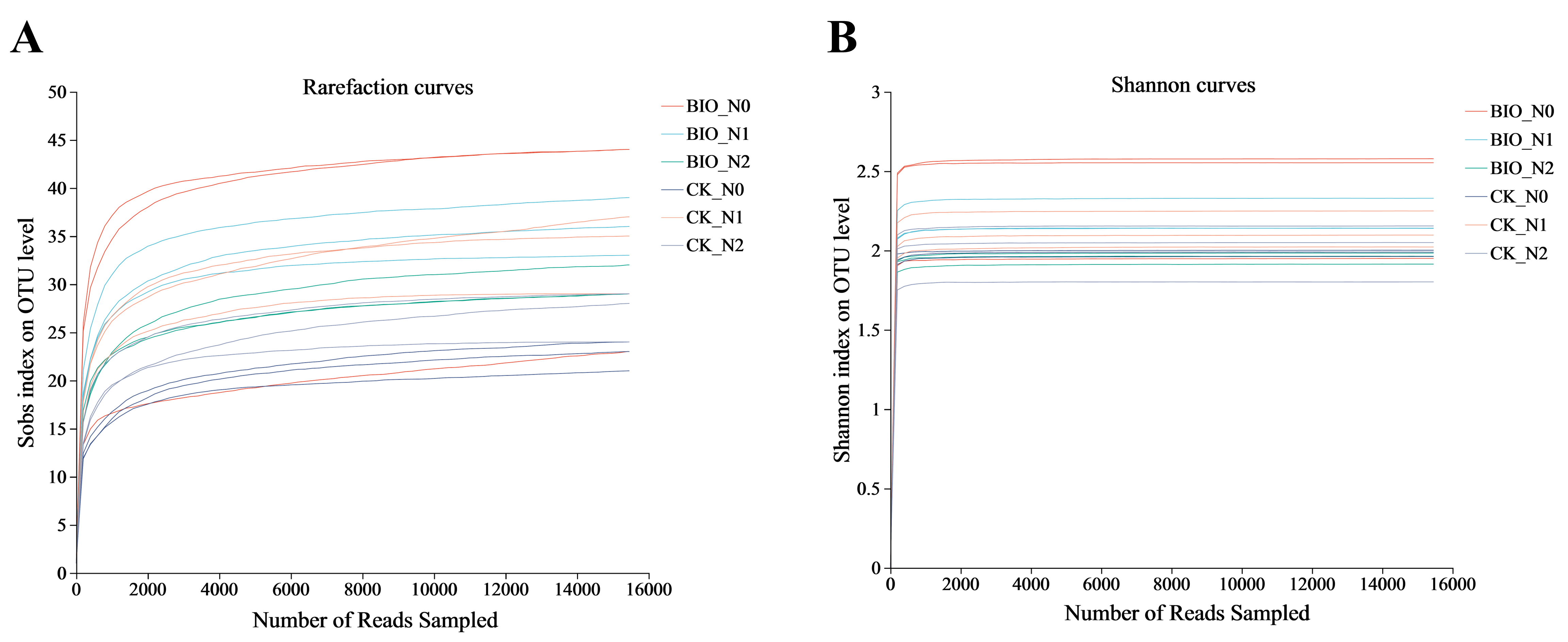

Supplement: SUPPLEMENTARY FIGURE S2 — Rarefaction and Shannon curves of rhizosphere microorganisms in different treatment groups at the OTU level. OTU, operational taxonomic unit; CK, control wherein the three nitrogen application levels were set up without bacteria; BIO, treatment wherein the three nitrogen application levels were set up with bacterial inoculation (Bacillus subtilis HG-15 + Bacillus velezensis JC-K3); N0, low nitrogen level; N1, normal nitrogen level; N2, high nitrogen level. [file Image_2.tif]

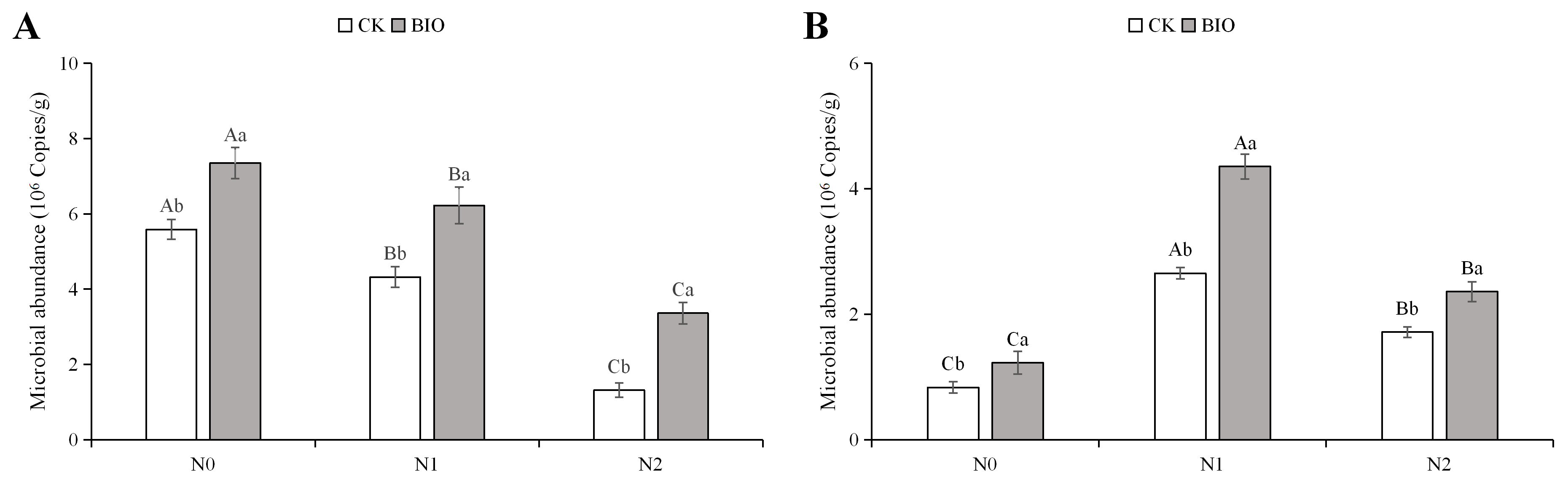

Supplement: SUPPLEMENTARY FIGURE S3 — Abundances of nitrogen-fixing bacteria (A) and arbuscular mycorrhizal fungi (B) in the apple rhizosphere soil of different treatment groups. [file Image_3.tif]

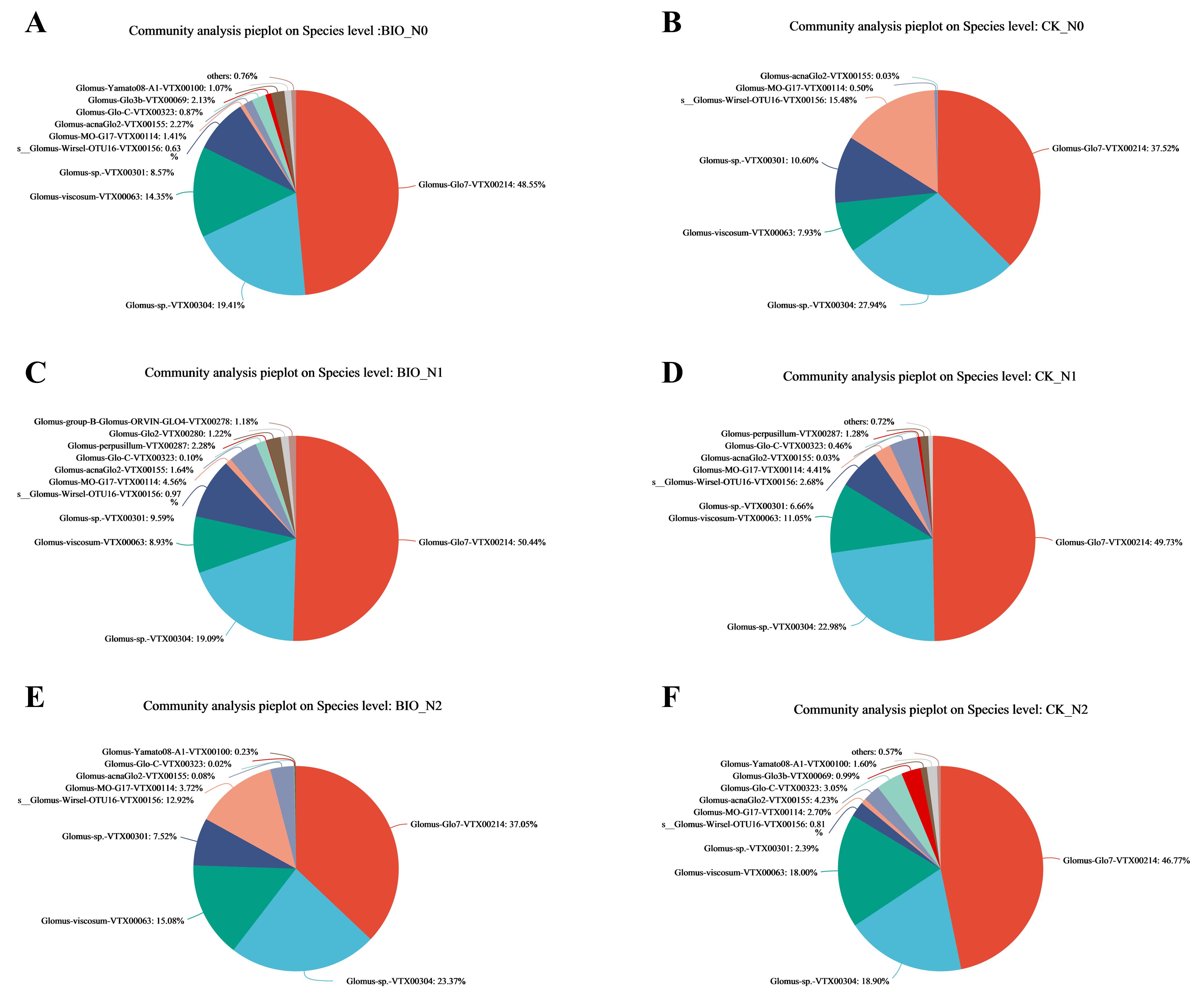

Supplement: SUPPLEMENTARY FIGURE S4 — Relative abundance (percentage) of AMF species in the apple rhizosphere soil from different treatment groups. [file Image_4.png]
